# Access to Alcohol Outlets, Alcohol Consumption and Mental Health

**DOI:** 10.1371/journal.pone.0053461

**Published:** 2013-01-16

**Authors:** Gavin Pereira, Lisa Wood, Sarah Foster, Fatima Haggar

**Affiliations:** 1 Yale Center for Perinatal, Pediatric, and Environmental Epidemiology, School of Public Health, Yale University, New Haven, Connecticut, United States of America; 2 Centre for the Built Environment and Health, School of Population Health, The University of Western Australia, Crawley, WA, Australia; 3 The Department of Surgery, The Ottawa Hospital Research Institute, The University of Ottawa, Ottawa, Canada; Catholic University of Sacred Heart of Rome, Italy

## Abstract

The objective of this study was to investigate residential exposure to alcohol outlets in relation to alcohol consumption and mental health morbidity (anxiety, stress, and depression). This was a cross-sectional study of 6,837 adults obtained from a population representative sample for the period 2006–2009 in Perth, Western Australia. The number of alcohol outlets was ascertained for a 1600 m service area surrounding the residential address. Zero-inflated negative binomial and logistic regression were used to assess associations with total alcohol consumption, harmful alcohol consumption (7–10 drinks containing 10 g of alcohol for men, 5–6 drinks for women) and medically diagnosed and hospital contacts (for anxiety, stress, and depression), respectively. The rate ratio for the number of days of harmful consumption of alcohol per month and the number of standard drinks of alcohol consumed per drinking day was 1.06 (95% CI: 1.02, 1.11) and 1.01 (95% CI: 1.00, 1.03) for each additional liquor store within a 1600 m service area, respectively. The odds ratio of hospital contact for anxiety, stress, or depression was 1.56 (95% CI: 0.98, 2.49) for those with a liquor store within the service area compared to those without. We observed strong evidence for a small association between residential exposure to liquor stores and harmful consumption of alcohol, and some support for a moderate-sized effect on hospital contacts for anxiety, stress, and depression.

## Introduction

Alcohol is the leading risk factor for disease burden in the Western Pacific and the Americas, and the second largest in Europe [1]. Globally the harmful use of alcohol is responsible for approximately 2.5 million deaths annually, yet there has been no decrease in worldwide per capita consumption [1]. Australia is no exception, with a recent report noting that despite public education efforts relating to the harmful effect of alcohol use, there was no observed decrease between 2001 and 2007 in the proportion of Australians drinking at ‘risky’ or ‘high-risk’ levels for long-term harm, which represented approximately 10% of the population [2]. In terms of consumption frequency, a 2007 national survey found that 40% of Australians drank alcohol weekly, and 8% drank on a daily basis [3]. In Western Australia, 39% of the population aged 14 years and over consume alcohol at levels that placed them at risk of short-term harm, and 11% reported drink levels that place them at risk of harm in the long-term [4]. Further, wholesale alcohol sales data indicate that the trend in per capita alcohol consumption in Western Australia is increasing [4].

The ready availability of alcohol is prominent among factors associated with higher levels of alcohol consumption and harm [5], [6], [7], with density of alcohol sales outlets the most frequently used measure of availability [7]. There are also community level consequences, with violence and crime among the most investigated community level outcomes to date. For instance, greater alcohol outlet density has been linked to higher rates of violence [8], violent crime [9], assaults [10] child maltreatment and physical abuse [11],[12], and homicides [13]. While most of the published studies to date have been conducted in the US, similar relationships between density and assault rates have been observed in Australia [14], [15], [16] [17]. Studies have also highlighted a relationship between outlet density and other traffic-related consequences, including drinking and driving, and riding with intoxicated drivers [18], alcohol-involved pedestrian collisions [19], traffic injury rates requiring hospitalisation [20] and alcohol-related crash fatalities [21].

Given growing research and public health interest in socio-economic disparities in health and health risk factors, a number of studies have investigated and documented associations between higher density of alcohol retail outlets and low SES or minority neighbourhoods [22], [23], [24], [25]. This parallels similar findings in the tobacco control literature which have reported higher densities of tobacco outlets in neighbourhoods characterized by social and economic disadvantage [26] [27] [28]. Ogneva-Himmelberger *et al* looked at both alcohol and tobacco outlet density within the same study and found that Massachusetts neighbourhoods with the highest levels of low-income populations and minorities tended to have the highest density of stores that sell tobacco and alcohol [29].

While there is a small but growing body of research investigating the relationship between alcohol outlet density and health, the focus has most often been on injury related harms [7] [30]. In a systematic review of studies undertaken by Popova *et al* in 2009, few of the 44 cross-sectional studies reviewed investigated non-injury related health outcomes [7]. Comparatively, there has been a paucity of studies into the relationship between other health morbidity and mortality and alcohol outlet density [15], with investigations pertaining to mental health outcomes a particular evidence gap. Livingston's [17] study is a rare exception, as it examined conditions related to the long term consumption of alcohol, including ‘mental and behavioural disorders’. Findings highlight a significant association between off-premises alcohol outlets and hospital admission rates for alcohol use disorders overall, however there was no separate reporting for mental health.

The potential impact of excessive alcohol consumption on mental health is widely accepted, with the majority of alcohol-related disease burden due to neuropsychiatric disorders, which include alcohol use disorders and depression [1]. The relationship between alcohol and mental health is also bi-directional, with evidence suggesting that among individuals more predisposed to harmful alcohol consumption are those prone to episodes of depression, anxiety and stress [31], [32]. However, there is a dearth of studies that have directly investigated individuals' access to alcohol outlets in relation to both their alcohol consumption and mental health disorders.

The aim of this study was to examine the effect of residential access to alcohol outlets on alcohol consumption, and determine whether elevated outlet density is associated with greater prevalence of mental health morbidity (anxiety, stress and depression).

## Methods

### Ethics approval

The Western Australian Department of Health obtained participant consent for use of their survey information for research purposes and for consent to linkage with other health data held by the department. The Western Australian Department of Health was responsible for obtaining ethics approval of the consent procedure. The authors obtained approval from the Human Research Ethics Committees of the Western Australian Department of Health and The University of Western Australia (#2010/1) to obtain and use this data for the research undertaken in this study. This research conforms to the ethical principles for medical research of the Declaration of Helsinki.

### Study design and participants

*A* cross-sectional study design was used to examine the association between individual-level alcohol outlet density and participants': (1) alcohol consumption; and (2) mental health morbidity. The sample comprised 6,837 adults aged 18 and over, who completed the Western Australian Health and Wellbeing Surveillance System (HWSS) Survey between 2006 and 2009 and were residents of the Perth metropolitan area. This monthly computer-assisted telephone interview was administered by the Western Australian Department of Health and responses were obtained for a stratified random sample of the state population (N = 1,959,088; 2006 Census).

#### Liquor store locations

The geocoded locations of off-premises alcohol retail establishments (referred to hereafter as liquor stores) were obtained from SENSIS Pty Ltd for 2005 and 2007. The 2005 SENSIS dataset was used for participants surveyed for the HWSS in the first six months of 2006. The 2007 SENSIS dataset was used for other participants. Each participant's residential address was also geocoded, and the number of liquor stores was calculated for their individual 1600 m road network distance service area [33]. The 1600 m service area represents the neighbourhood environment, and is based on the distance a participant could walk to and back (i.e., a return trip) at moderate to vigorous intensity pace, within 30 minutes. Liquor store location data were matched to the year participants' completed the HWSS survey. We focused on liquor stores (i.e., alcohol sales to be consumed away from the establishment), as previous research has highlighted a stronger association between postcode-level packaged alcohol sales and chronic alcohol-caused hospitalisation, than for establishments where alcohol is consumed on-premises [17].

#### Mental health outcomes

We examined mental health morbidity outcomes from two sources. Self-report of prior medical diagnosis with anxiety, stress and depression was obtained from the HWSS Survey and analysed as a single outcome. Hospital admissions, outpatient contacts and emergency mental health contacts were obtained from the Department of Health for all participants who granted permission for data linkage (74%). Anxiety, stress and depression were identified from hospital records as a primary diagnosis coded 300, 309 and 311 according to the International Classification of Diseases 09 (ICD-09) and F30-99 according to ICD-10-CM. Participants were considered to have been hospitalised for anxiety, stress or depression if the admission occurred within a three year window centred on the year that the participant completed the HSWW Survey.

#### Alcohol consumption outcomes

The number of standard drinks of alcohol consumed per drinking day and the number of days of harmful consumption of alcohol in the past four weeks were obtained from the HWSS Survey. A standard drink was defined as a drink containing 10 grams of alcohol. Harmful consumption was defined by the Department of Health in this survey as 7 to 10 standard drinks in a day for men and 5 to 6 standard drinks in a day for women. These correspond to the definition of ‘risky drinking’ as specified in the National Health and Medical Research Council Australian Alcohol Guidelines, [34],[35].

### Statistical analysis and adjustment

Analyses adjusted for participant age, sex, education and household income (obtained from the HWSS Survey). For the mental health outcomes, logistic regression was used to calculate Odds Ratios (OR) comparing participants with a liquor store within the service area to those without a liquor store in the service area. For the alcohol consumption outcomes, zero-inflated negative binomial regression was used to calculate rate ratios (RR) per additional liquor store in the service area. A zero-inflated negative binomial model was selected to account for both over-dispersion and excess zeroes (abstainers from alcohol). The choice of model was confirmed using Vuong's test [36]. Participant age, sex, education and household income were used as predictors in the logit component of the zero-inflated negative binomial model.

District-level socioeconomic status was ascertained using the socioeconomic index for areas (SEIFA) score obtained from the Australian Bureau of Statistics [37]. The SEIFA score is based on the census collection district of the participant residence and is an area-based index of relative socioeconomic advantage and disadvantage. Census collection districts contain an average of 250 dwellings. Lower SEIFA scores indicate relative socioeconomic disadvantage and higher scores indicate relative advantage. The SEIFA index has a national mean of 1000 and a standard deviation of 100. The number of liquor stores within each census collection district was summed and the mean numbers of liquor stores were calculated and compared across each SEIFA tertile. All analyses were conducted in SAS v9.2.

## Results

The study population consisted of 6,837 adult participants, resident in the Perth metropolitan area of Western Australia (Table 1). There were 100 (1%) adults with a hospital contact for anxiety, stress or depression and 957 (14%) adults that reported a prior medical diagnosis with these conditions. The mean number of standard drinks consumed on a drinking day was 1.95 (standard deviation (SD) 2.27) and the mean number of days of harmful drinking in the preceding four weeks was 0.89 (SD 2.99). The mean number of liquor stores within a 1600 m service area was 1.39 (SD 1.79).

**Table 1:** Study characteristics of adults resident in Perth, Western Australia, who responded to the Health and Wellbeing Survey 2006–2009 for the study cohort that consented to data linkage (N = 6,837), and the non-linkable population who did not consent to data linkage and subsequently excluded.

	Study PopulationN = 6,837	Non-linkable PopulationN = 2,409
	**Mean (SD)**	**Mean (SD)**
**Number of standard drinks of alcohol consumed per drinking day** a	1.95 (2.27)	1.79 (2.07)
**Number of days of harmful consumption of alcohol in the past four weeks** b	0.89 (2.99)	0.60 (2.43)
**Number of liquor stores within a 1600 m service area of the residential address** c	1.39 (1.79)	NA
	**N (%)**	**N (%)**
**Anxiety, stress, depression**		
Ever diagnosed	957 (14)	351 (15)
Hospital contact	100 (1)	NA
**Sex**		
Female	4,051 (59)	1,520 (63)
Male	2,786 (41)	889 (37)
**Age group**		
18–24 years	505 (7)	161 (7)
25–34 years	566 (8)	260 (11)
35–44 years	1,040 (15)	390 (16)
45–54 years	1,186 (17)	489 (20)
55–64 years	1,474 (22)	530 (22)
65 years and over	2,066 (30)	579 (24)
**Highest attained level of education**		
Less than year 10	548 (8)	211 (9)
Year 10 or 11	1,134 (17)	385 (16)
Year 12	838 (12)	342 (14)
Trade qualification	2,764 (40)	852 (35)
Tertiary degree	1,528 (22)	605 (25)
**Household Income**		
Less than $20,000	1,063 (16)	287 (12)
$20,001–40,000	1,325 (19)	350 (15)
$40,001–$60,000	878 (13)	254 (11)
$60,001–$80,000	878 (13)	262 (11)
More than $80,000	2,000 (29)	555 (23)

a . A standard drink is defined as any drink containing 10 grams of alcohol.

b . Harmful consumption was defined by the Department of Health in this survey as 7–10 standard drinks in a day for men, and 5–6 standard drinks in a day for women.

c . The service area was defined as the area accessible to a distance of 1600 m from the residential address along the road network.

NA. No data available because the participants did not agree to linkage of the HWSS survey responses to either hospital records or to environmental data (liquor store locations).

The “non-linkable population” consisted of 26% (N = 2,409) of the total population who did not provide consent to link their HWSS survey responses to hospital records or environmental variables (liquor store locations) using their residential address (Table 1). Compared to the study population the non-linkable population enaged in fewer days of harmful alcohol consumption, had a greater proportion of female participants and had a lower proportion of participants in the 65+ age category. There was also indication that they had attained a higher level of education, with proportionally more participants educated to year 12 or with a tertiary degree, and fewer with a trade qualification.

### Association between residential exposure to liquor stores and alcohol consumption

After adjustment for age, sex, education and household income, there was marginal support (p = 0.054) for an association between the number of standard drinks of alcohol consumed per drinking day and the number of liquor stores within the service area; RR 1.01 (95% CI: 1.00, 1.03) (Table 2). Evidence was stronger (p = 0.006) for an association with harmful consumption of alcohol in the past four weeks, with harmful alcohol consumption increasing by 6% for every additional liquor store within the 1600 m neighbourhood; RR 1.06 (95% CI: 1.02, 1.11).

**Table 2:** Rate ratios (RR) and 95% confidence intervals (CI) of total and harmful alcohol consumption for increases in the number of liquor stores within a 1600 m service area about the residential address.

	Unadjusted		Adjusted^a^	
	RR (95% CI)	p-value	RR (95% CI)	p-value
*Number of standard drinks of alcohol consumed per drinking day* b				
Per liquor store in the service area	1.00 (0.99, 1.02)	0.617	1.01 (1.00, 1.03)	0.054
*Number of days of harmful consumption of alcohol in the past four weeks* c				
Per liquor store in the service area	1.06 (1.02, 1.10)	0.007	1.06 (1.02, 1.11)	0.006

a . Adjusted for age, sex, income and education.

b . The standard drink is defined as any drink containing 10 grams of alcohol.

c . Harmful consumption was defined by the Department of Health in this survey as 7–10 standard drinks in a day for men, and 5–6 standard drinks in a day for women.

### Association between residential exposure to liquor stores and mental health morbidity

There was negligible evidence of an association between self-reported prior medical diagnosis with a mental health disorder (anxiety, stress or depression) and presence of a liquor store in the 1600 m service area (Table 3). However, the unadjusted odds ratio for hospital contact with a mental health disorder was 1.58 (95% CI: 1.04, 2.41), comparing participants with at least one liquor store in the service area to those without a liquor store. The strength of the evidence of this effect attenuated after adjustment for age, sex, education and household income (from p = 0.032 to 0.059), and the odds ratio attenuated slightly 1.56 (95% CI: 0.98, 2.49).

**Table 3:** Odds ratios (OR) and 95% confidence intervals (CI) of mental health morbidity (anxiety, stress and depression) for presence of a liquor stores within a 1600 m service area about the residential address.

	Unadjusted		Adjusted^a^	
	OR (95% CI)	p-value	OR (95% CI)	p-value
*Hospital admission, outpatient or emergency contact for anxiety, stress or depression* b				
No liquor stores	1	Ref	1	ref
At least 1 liquor store	1.58 (1.04, 2.41)	0.032	1.56 (0.98, 2.49)	0.059
*Self-reported prior medical diagnosis of anxiety, stress or depression*				
No liquor stores	1	Ref	1	ref
At least 1 liquor store	1.07 (0.93, 1.23)	0.336	1.07 (0.92, 1.24)	0.400

a . Adjusted for age, sex, income and education.

b . Hospital contacts within a 3-year period centred on the year of completion of the Health and Wellbeing Survey.

### Association between prevalence of liquor stores and district-level socioeconomic status

The mean number of *liquor stores* in census collection districts with the lowest SEIFA values (i.e., lowest tertile) was 1.62 (95% CI: 1.42, 1.82). For census collection districts with moderate (middle tertile) and high SEIFA values (highest tertile), the mean number of liquor stores was 1.45 (95% CI: 1.25, 1.64) and 1.55 (95% CI: 1.35, 1.75).

## Discussion

In this study, we investigated the relationship between liquor store density and alcohol consumption, and between density and risk of hospital admissions for anxiety, stress and depression. These associations were observed after accounting for socio-demographics, including correlates of socioeconomic status. The number of liquor stores in the neighbourhood was weakly associated with total alcohol consumption, but more strongly associated with harmful alcohol consumption. Each additional liquor store in the neighbourhood was associated with an increase by 1% in the mean number of standard drinks of alcohol consumed per drinking day and by 6% in the mean number of days of harmful consumption of alcohol. The public health impact of liquor stores on alcohol consumption depends not only on the magnitude of these risk estimates, but also the distribution of exposure across the population. Therefore, if our results represent a true effect, the 1% increase in the mean number of standard drinks would affect the 83% [3] of the national population (aged 14+) who drink, and the 10% [3] of the national population (aged 14+) who already drink at levels that place their health at risk.

Our study also sought to address a gap in the literature by specifically investigating the relationship between liquor store density and mental health, with a data set that enabled us to look at this at the individual level. Further information as to the pathways between liquor store density and mental health is provided in the Supporting Information (Text S1). We observed that the odds of hospital contact for anxiety, stress or depression was 56% greater among participants with a liquor store within the neighbourhood compared to those without. The statistical non-significance of this adjusted effect estimate was possibly a result of reduced statistical power arising from the rarity of a hospital contact for these mental health conditions and subsequent small number of events (N = 100, 1%).

These results are compelling in terms of public health advocacy and policy, and support arguments to limit the number of liquor stores in a given area as a means to limit (i.e. further regulate) alcohol availability and minimise alcohol-related harm [7]. In Western Australia, decisions to grant new liquor licenses in a given area require consideration of the public interest [38]. Demonstration of an association between presence of liquor stores, harmful alcohol consumption and mental health morbidity motivates progressing from the economic-centric view of the ‘public interest’ to better incorporate the public *health* interest. The WHO Global Strategy to Reduce the Harmful Use of Alcohol lists regulation of alcohol availability as an important way to reduce the general level of harmful use of alcohol, and listed regulating the number and location of liquor stores as a possible option [39]. Moreover, as the mean number of liquor stores was slightly higher in areas of with a lower socioeconomic index, the findings of this study are particularly relevant for policy-related interventions to level socioeconomic gradients in outlet density. A limitation of the cross-sectional nature of this study was that we could not discern whether the association between liquor stores and alcohol consumption was attributable to (i) the presence of the outlets promoting increased consumption, or (ii) whether the outlets were attracted to the more profitable (possibly more socioeconomically disadvantaged) neighborhoods. However, closer scrutiny of new liquor licenses is warranted in either case.

While one of the strengths of this study was the inclusion of objective hospital health data, hospital admissions clearly under-represents the overall burden of mental health as it does not capture diagnoses of depression, anxiety or other mental health problems made through a GP, nor those that may go undetected. Although this issue cannot be completely resolved we also included self-reported doctor diagnoses to supplement outcome assessment. A further limitation of this study was that although we could ascertain the level of alcohol consumption, we did not have information on the location where this alcohol was purchased and we recommend this information be obtained in future cohort studies. For ecological studies, it has been demonstrated that use of alcohol sales data might be an appropriate proxy [14]. However, it is likely that such misclassification would have attenuated our effect-estimates. A related limitation of this study was the lack of adjustment for locations that sold alcohol for on-premises consumption considered in previous studies [14], [17], which might have been geographically clustered with locations that sold alcohol for off-premises consumption. However, Livingston observed much stronger associations between chronic alcohol-caused hospitalization and locations of off-premises outlets than on-premises outlets [17]. It is also possible that further attenuation of effect-estimates would have been introduced due to reduced time relevance of the liquor store locations dataset for participants surveyed for the HWSS at the end of the study period in 2009.

## Conclusions

Participants with greater access to liquor stores were more likely to consume alcohol at harmful levels and to have had a hospital contact for anxiety, stress or depression. The findings underscore the importance of policy approaches that limit both the number of liquor store licences and the geographic density of outlets as a means to improve mental health and reduce other alcohol related harm. We proposed a range of explanatory pathways to advance understanding of how proximate access to alcohol sales outlets might impact drinking levels and residents' mental health, ranging from availability, affordability and normative cues, through to the impact of liquor stores on local amenity and social capital. Further research is needed however to test and better understand these pathways, with a view to informing policy measures to reduce the negative effects of ubiquitous alcohol access in local communities.

## Supporting Information

Text S1 **Potential pathways between alcohol outlet density and mental health.**
(DOCX)
