# Supplementary material for: Access to Alcohol Outlets, Alcohol Consumption and Mental Health
Source: PLoS One. 2013 Jan 16;8(1):e53461. doi: 10.1371/journal.pone.0053461 (PMC3547008; doi:10.1371/journal.pone.0053461)
Supplement: Text S1 — Potential pathways between alcohol outlet density and mental health. (DOCX) [file pone.0053461.s001.docx]

Supporting Information (Text S1) for

*Access to alcohol outlets, alcohol consumption and mental health*

Gavin Pereira^1, 2 *^, Lisa Wood^2^, Sarah Foster^2^, Fatima Haggar^3^

1 Yale Center for Perinatal, Pediatric, and Environmental Epidemiology, School of Public Health, Yale University, New Haven, Connecticut, USA. E: [gavin.pereira@yale.edu](mailto:gavin.pereira@yale.edu). P: +1 (203) 764-9767.

2 Centre for the Built Environment and Health, School of Population Health, The University of Western Australia, M707, 35 Stirling Highway, Crawley WA 6009, Australia.

3 The Department of Surgery, The Ottawa Hospital Research Institute, The University of Ottawa, Ottawa, Canada

**Potential pathways between alcohol outlet density and mental health**

Further research is needed to understand the pathways through which alcohol outlet density impacts on mental health, and the very nature of mental health suggests that the trajectory is likely complex and multi-faceted. One of the criticisms of the alcohol density research to date is that the primary focus has been on the statistical relationship, without the development of suitable theory regarding how these relationships emerge in community settings[[1](#_ENREF_1)]. In response, we posit five potential pathways between density and mental health that merit further investigation:

**Availability and use of alcohol**. The density of alcohol outlets is a marker of proximate availability. While not all studies to date have reported a relationship between density and alcohol use [[2](#_ENREF_2)], there is a growing body of research associating higher levels of alcohol availability with higher alcohol consumption in both adults ([[3](#_ENREF_3)] and adolescents [[4](#_ENREF_4)] and in turn, higher levels of alcohol-related harm [[3](#_ENREF_3)]. Conversely, Scribner *et al* found that longer average distance between the nearest alcohol outlet and residents was significantly associated with a lower quantity of alcohol consumption[[5](#_ENREF_5)]. Our findings lend support to these results. However, we observed that the presence of liquor stores was most relevant for high risk alcohol consumption than general alcohol consumption, indicating that if the pathway is mediated by proximate availability; those predisposed to high risk drinking are most vulnerable.

**Affordability.** It has also been hypothesised that increased alcohol outlet density can impact on alcohol related harm via an ‘economic’ pathway whereby “increasing the number of alcohol outlets reduces the real cost of alcohol (incorporating the convenience of making a purchase, along with the monetary price), thus increasing consumption and related problems”[[6](#_ENREF_6)]. Similarly, it has been suggested that alcohol availability, particularly outlet density, can increase market competition and thereby make discounted alcohol more available to those already predisposed to harmful alcohol consumption [[7](#_ENREF_7)].

**Amenity effect**. Coined by Livingston et al, amenity effect refers to the way in which the presence of alcohol outlets ‘can influence the quality and characteristics of surrounds within the local community’ [[8](#_ENREF_8)]. In Australia, Donnelly *et al* found that people who lived in closer proximity to liquor outlets were more likely to report neighbourhood problems such as drunkenness and property damage, even after controlling for socio-demographic factors [[9](#_ENREF_9)]. Other examples of negative amenity effects include visible public intoxication, violence, street disturbances and other social problems[[3](#_ENREF_3)],[[8](#_ENREF_8)]. In turn, this can adversely affect mental health in a number of ways, via the elevation of environmental stressors, and the impact on feelings of safety, anxiety and sense of community) [[10](#_ENREF_10),[11](#_ENREF_11),[12](#_ENREF_12)].

**Visual and normative cues**. There are visual and normative cues embodied in the presence of alcohol outlets (on or off premise) within a neighbourhood, and the ‘quantity’ of this is influenced in part by density. Such cues include the social modelling of drinking behaviors, visibility of advertising and promotions, and incidental purchase behaviour[[1](#_ENREF_1)].In terms of mental health, such visual cues could normalise or reinforce alcohol as an option for coping with stress or difficult times or contribute to increased use and in turn health-related harms. This ‘pathway’ between density and mental health may not be direct, yet merits further investigation as one of the unintended and less researched consequences of alcohol density. Additionally, while the nexus between availability and tobacco advertising has been broken in Australia through advertising bans, this is not yet the case with alcohol. As suggested in a recent review by Bryden *et al* [[2](#_ENREF_2)] greater exposure to alcohol advertising in a community (e.g., billboards, signage) may be associated with higher levels of drinking or with the likelihood of adolescents ever having tried alcohol.

**Social capital and mental health**. The connection between social capital and alcohol density has recently been explored by Theall et al (2009) [[13](#_ENREF_13)]. Unlike some pathways outlined above, they contend that off premise alcohol outlets could exert either a positive or negative influence on social capital, and the direction of the relationship depends on the role of the outlet within the community. They suggest that, “in the case of network expansion or contraction, a well-run outlet could provide a meeting place for residents to expand their social interactions; alternatively, it could lead to network contraction if the outlet does not cater to local neighborhood residents and instead threatens residents with loud noise, unruly patrons, trash, late hours of operation, and other incivilities that have been linked to off-premise alcohol outlets (e.g., crime)”[[13](#_ENREF_13)].

That the influence can be positive or negative is congruent with the conceptual nature of social capital [[14](#_ENREF_14)] and with other research into the role that elements of the built environment can play in growing or depleting the social capital stocks of a community [[15](#_ENREF_15)] [[16](#_ENREF_16)]. As conceptualised by Theall *et al*, there is an interactive relationship between alcohol outlets and social networks at the neighbourhood level, and this can contribute to social capital which can in turn influence health outcomes. Neither mental health nor other specific health outcomes were explored by Theall *et al* (2009), but there is a growing body of other research that underscores the link between mental health and social capital [[17](#_ENREF_17),[18](#_ENREF_18),[19](#_ENREF_19)]. Our findings motivate further study in this population of Perth, Western Australia, commencing with an investigation of the role of social capital as an intermediate between alcohol outlets, alcohol consumption and mental health.

**References**

1. Gruenwald PJ (2007) The spatial ecology of alcohol problems: niche theory and assortative drinking. Addiction 102: 870-878.

2. Bryden A, Roberts B, McKee M, Petticrew M (2012) A systematic review of the influence on alcohol use of community level availability and marketing of alcohol. Health & Place 18: 349-357.

3. Popova S, Giesbrecht N, Bekmuradov D, Patra J (2009) Hours and Days of Sale and Density of Alcohol Outlets: Impacts on Alcohol Consumption and Damage: A Systematic Review. Alcohol and Alcoholism 44: 500-516.

4. Truong KD, Strum R (2009) Alcohol Environments and Disparities in Exposure Associated With Adolescent Drinking in California American Journal of Public Health 99: 264-270.

5. Scribner R (1999) Alcohol availability and homicide in New Orleans: conceptual considerations for small area analysis of the effect of alcohol outlet density. Journal of studies on alcohol 60: 310.

6. Livingston M (2011) Alcohol outlet density and harm: Comparing the impacts on violence and chronic harms. Drug and Alcohol Review 30: 515-523.

7. Gruenewald PJ, Millar AB, Roeper P (1996) Access to alcohol: geography and prevention for local communities. Alcohol Health and Research World 20: 244-251.

8. Livingston M, Chikritzhs T, Room R (2007) Changing the density of alcohol outlets to reduce alcohol-related problems. Drug and Alcohol Review 26: 557-566.

9. Donnelly N, Poynton S, Weatherburn D, Bamford E, Nottage J (2006) Liquor outlet concentrations and alcohol-related neighbourhood problems. Alcohol Studies Bulletin. Available: <http://www.lawlink.nsw.gov.au/lawlink/bocsar/ll_bocsar.nsf/vwfiles/ab08.pdf/$file/ab08.pdf>. Accessed 15 March 2012.

10. Evans G (2003) The built environment and mental health. Journal of Urban Health 80: 536-555.

11. Halpern D (1995) More than Bricks and Mortar? Mental Health and the Built Environment. London: Taylor & Francis Ltd. 240 p.

12. Lorenc T, Clayton S, Neary D, Whitehead M, Petticrew M, et al. (2012) Crime, fear of crime, environment, and mental health and wellbeing: Mapping review of theories and causal pathways. Health & Place 18: 757-765.

13. Theall KP, Scribner R, Deborah C, Bulthenthal RN, Schonlau M, et al. (2009) Social capital and the neighborhood alcohol envrionment. Health & Place 15: 323-332.

14. Portes A (1998) Social capital: its origins and applications in modern sociology. Annual Review of Sociology 24: 1 - 24.

15. Sooman A, Macintyre S (1995) Health and Perceptions of the Local Environment in Socially Contrasting Neighbourhoods in Glasgow. Health and Place 1: 15-26.

16. Wood L, Giles-Corti B (2008) Is there a place for social capital in the psychology of health and place? Journal of Environmental Psychology 28: 154-163.

17. De Silva MJ, McKenzie K, Harpham T, Huttly SRA (2005) Social capital and mental illness: a systematic review. Journal of Epidemiology and Community Health 59: 619-627.

18. Almedom AM (2005) Social capital and mental health: An interdisciplinary review of primary evidence. Social Science & Medicine 61: 943-964.

19. Araya R, Dunstan F, Playle R, Thomas H, Palmer S, et al. (2006) Perceptions of social capital and the built environment and mental health. Social Science & Medicine 62: 3072-3083.
